# Immune cell infiltration and modulation of the blood-brain barrier in a guinea pig model of tuberculosis: Observations without evidence of bacterial dissemination to the brain

**DOI:** 10.1371/journal.pone.0307577

**Published:** 2024-12-31

**Authors:** Amanda S. Latham, Charlize E. Geer, David F. Ackart, Kristin N. Weninger, Chase C. Gross, Brendan K. Podell, Randall J. Basaraba, Julie A. Moreno

**Affiliations:** 1 Department of Environmental and Radiological Health Sciences, College of Veterinary Medicine and Biomedical Sciences, Colorado State University, Fort Collins, Colorado, United States of America; 2 Brain Research Center, Colorado State University, Fort Collins, Colorado, United States of America; 3 Department of Microbiology, Immunology and Pathology, College of Veterinary Medicine and Biomedical Sciences, Colorado State University, Fort Collins, Colorado, United States of America; 4 Department of Biomedical Science, College of Veterinary Medicine and Biomedical Sciences, Colorado State University, Fort Collins, Colorado, United States of America; 5 Mycobacteria Research Laboratories, Department of Microbiology, Immunology and Pathology, College of Veterinary Medicine and Biomedical Sciences, Colorado State University, Fort Collins, Colorado, United States of America; 6 Center for Healthy Aging, Colorado State University, Fort Collins, Colorado, United States of America; Centenary Institute, AUSTRALIA

## Abstract

Tuberculosis (TB), caused by *Mycobacterium tuberculosis* (Mtb) infection, is a chronic inflammatory disease. Although typically associated with inflammation of the lungs and other peripheral tissues, increasing evidence has uncovered neurological consequences attributable to Mtb infection. These include deficits in memory and cognition, increased risk for neurodegenerative disease, and progressive neuropathology. Although the neurological effects of the disease, without CNS infection, have been characterized, the mechanism of neurotoxicity is unknown. We hypothesized that alterations to the blood-brain barrier (BBB) allows peripheral immune cells to enter the brain, initiating a neuroinflammatory response. To test this hypothesis, guinea pigs were exposed by aerosol to a laboratory and a clinical Mtb strain for 15 days. Following Mtb infection, proteins critical to BBB function, including claudin V and collagen IV, are modulated without evidence of bacterial dissemination to the brain. This is correlated with increased contact of astrocytic processes to vessels in the brain, as well as increased expression of the water channel protein aquaporin 4 (AQP4) on endfeet. Upon further investigation, we discovered the potential role of glial reactivity, which is increased following infection with both bacterial strains, in the progression of BBB changes and, ultimately, the permeability of peripheral immune cells into the brain. Through these data, we have obtained a preliminary understanding of the mechanisms of cellular stress in the brain following pulmonary Mtb infection which should be further investigated in future studies.

## Introduction

Tuberculosis (TB) is catastrophic disease, caused by infection with *Mycobacterium tuberculosis* (Mtb), that affects approximately 1.7 billion people across the globe [1]. An estimated 10 million new cases of active disease occur each year worldwide, which is only worsening despite global strategies for disease elimination [2, 3]. Thus, it is crucial that research be performed to not only interfere with the progression of disease, but to better understand the long-term consequences of infection. Although much is known about the immune response to Mtb in peripheral tissues, little information exists describing the neurological effects of TB, despite published epidemiological data detailing long-term cognitive changes associated with the disease. These studies show that diagnosed TB is correlated with neurological deficits, including memory loss and decreased cognitive functioning [4]. TB also predisposes individuals to neurodegenerative disease, including Parkinson’s Disease (PD) and dementia [5–7]. Interestingly, these documented findings present in human patients who are not diagnosed with central nervous system (CNS) infection or tuberculosis meningitis (TBM), emphasizing the connection between pulmonary infection and the brain. Unfortunately, while it is established that TB can increase risk for disease, the complete neurological consequences, and pathological mechanisms driving them, remain unclear. Our previous findings help to characterize the neuropathologies that occur during the progression of disease in an established *in vivo* model of TB. In our model of peripheral disease without evidence of CNS infection, we correlated behavior changes to the presence of neuropathologies identified in neurological disorders, including glial inflammation, the accumulation of misfolded and aggregated proteins, and neuronal loss in the hippocampus [8].

Glial inflammation is considered one of the earliest detectable abnormalities in neurological disease. Glia, astrocytes and microglia, can polarize from neuroprotective phenotypes into pro-inflammatory, neurotoxic ones. Although this response acutely helps to restore the microenvironment in response to stress, prolonged cellular activation compromises the brain over time. Microglia, which function as the resident immune cell of the brain, utilize their phagocytic capacity to eliminate pathogens, protein aggregates, and apoptotic cellular debris. Altogether, these actions aid in maintaining neuronal synapses. Microglial activation occurs following the recognition of antigens or cytokines and chemokines. These include tumor necrosis factor (TNF), interleukin-6 (IL-6), and interleukin-1b (IL-1b) [9]. Once in a reactive state, these cells actively secrete pro-inflammatory mediators that stimulate nearby cells; they are the primary producers of complement component 1, subcomponent q (C1q), interleukin 1α (Il-1α), and TNF. This molecular combination is one well-established method of astrocyte activation [10]. Once stimulated, neurotoxic astrocytes no longer perform supportive functions, including regulating neurogenesis and maintaining neurotransmitter levels. Similar to microglia, they contribute to pro-inflammation and produce complement proteins involved in opsonization and immune cell recruitment. Subsequently, colocalization of complement 3 (C3) in astrocytic cell bodies is an indication of cellular reactivity, along with others, such as increased expression of glial fibrillary acidic protein (GFAP) and S100 calcium-binding protein β (S100β) [11, 12]. Furthermore, astrocytes, through their terminal processes or “endfeet”, play an essential role in blood-brain barrier (BBB) function.

In addition to glial inflammation, reduced integrity of the BBB is found in numerous neurological disorders, including stroke, CNS infections, Alzheimer’s Disease (AD), and PD, among others [13, 14]. The brain is a highly controlled microenvironment composed of multiple protective mechanisms that separate the CNS from the peripheral circulation. The BBB uses both physical and metabolic methods to maintain the physiological environment of the brain. This barrier consists of molecular components, including the glycocalyx and basement membrane, as well as various cellular players. The cellular components, endothelial cells (ECs), pericytes (PCs), and astrocyte endfeet, interact with one another to form the “neurovascular unit” that limits permeation of molecules and cellular traffic to and from the brain [15]. Although located throughout the body, the ECs forming vessels within the CNS are unique; they lack fenestrations and form extensive tight junctions (TJs) between cells [16]. The main functional elements of TJs are transmembrane claudin proteins, which tighten the paracellular cleft, form pores, and contribute to the maturation of this barrier. One claudin protein, in particular, claudin V, is the most highly expressed protein of this type in the brain. It is a critical constituent that acts as a “sealing” protein to maintain the tightness of TJs, preventing small molecules (< 800 Da) from entering the brain [17–19]. Altogether, TJs form a physical barrier that inhibits cells and other biomolecules from entering the brain parenchyma. Compromising components of these TJs, as documented during injury and disease, leads to increased paracellular solute leak [20].

On the parenchymal side of the BBB sits a basement membrane, an extracellular matrix primarily composed of collagen, laminin, nidogen, and perlecan. Collagen IV is the most abundant protein in the basement membrane and is critical for maintaining the integrity and function of the BBB [21]. Expression of collagen in microvessels is unique in that both downregulation and upregulation of this protein is implicated in disease. In murine models, mutations in alpha chains of collagen IV or complete loss of the protein results in pathology similar to porencephaly and small vessel disease, leading to brain hemorrhage [22, 23]. In models of bacterial meningitis and herpes-simplex virus encephalitis, the extent of BBB breakdown and subsequent cortical injury is directly correlated with the degree of collagen IV degradation by matrix metalloproteinases (MMPs) [24, 25]. Alternatively, collagen accumulation is associated with BBB leak and cognitive impairment in models of hypertension and ischemia [26]. Increased collagen IV content is also seen in the brains of human patients diagnosed with AD [27]. This variability in disease-associated collagen expression showcases the complexity of this protein’s function in the BBB.

Critically, the BBB maintains the chemical composition of the brain, which is necessary for proper neuronal function. Such maintenance is performed, in part, by astrocytes. Astrocytic endfeet physically contact the endothelial cells and pericytes that form the vasculature, and express proteins like aquaporin 4 (AQP4), a water transport protein. These proteins allow glial cells to regulate ion concentrations within the brain. Astrocytes also modulate neuronal activity and cerebral blood flow by controlling intracellular calcium levels in their endfeet, resulting in vasodilation and vasoconstriction [28]. The physical connection between astrocytic endfeet and vessels is highly implicated in neuroinflammation. Disruption of astrocytic endfeet and their proteins, in addition to other components of the BBB, results in neuroinflammation and cellular infiltration. Separating endfeet from the vasculature disrupts tight junction proteins, including claudins, and leads to biomolecular leak into the brain [29]. Loss of AQP4 in astrocytic endfeet is seen in experimental autoimmune encephalomyelitis and neuromyelitis optica, correlating to reduced BBB function. Overall, decline in astrocyte endfeet and their transporters disrupts ion equilibrium within the brain. This alters synaptic transmission and neuronal excitability which, ultimately, results in the loss of neuronal viability. Reactive astrocytes also secrete inflammatory mediators and reactive species, instead of supportive factors, that further reduce the integrity of the BBB and damage the cells native to the CNS.

Astrocytes develop and maintain the BBB through the release of growth factors like glial cell line-derived neurotrophic factor (GDNF) [30, 31]. In response to inflammation, astrocytes stimulate the formation of tight junctions, through other secreted factors including angiopoietin-1 (ANG1) and sonic hedgehog (SHH), to limit permeation of immune cells into the brain [31–34]. Despite evidence that astrocytes produce BBB stimulating molecules, the exact role of these cells in maintaining barrier integrity is contradictory. Several studies conclude that astrocyte ablation does not alter BBB structure or result in microvascular leak [35, 36]. Others suggest that loss of astrocytes compromises tight junctions, allowing molecules of various sizes to extravasate into the brain [37]. *In vitro* models of the BBB support this, as co-cultures of astrocytes and endothelial cells increase the formation of tight junctions compared to endothelial monocultures alone [38].

Due to the importance of various structural proteins in BBB function, their reduced expression is often used as a determination of barrier integrity, as seen in previous studies. This loss can occur through multiple pathophysiological mechanisms, including oxidative stress and activation of cytokine-mediated intracellular signaling. Overall, these mechanisms disrupt tight junctions and alter molecular transport, leading to increased leakage of biomolecules and infiltration of immune cells from the periphery. This allows an influx of neurotoxins, microbial components, inflammatory mediators, or activated cells into the brain, promoting an inflammatory brain phenotype that is chronically damaging [39]. Subsequently, BBB dysfunction is considered an early and significant event in the pathogenesis of neurological disease. The integrity of the BBB during CNS infection has historically been of interest, as bacteria and viruses can alter the BBB to facilitate entry into the brain [40]. In an *in vitro* co-culture model of the BBB, Mtb exposure increases permeability by stimulating MMP-dependent breakdown of tight junction proteins [41]. In patients with TBM, the most common form of CNS Mtb infection, BBB permeability is significantly higher [42].

More recently, BBB damage has been implicated as a critical pathology in AD and PD. In AD, vascular permeability, which has been identified in human patients, is correlated with disease onset and severity [43]. Similarly, BBB damage and leaking has been identified in human PD patient brains [44]. Not only is barrier dysfunction implicated in CNS disease, but peripheral inflammation can also mediate BBB changes. Murine models of AD increase leakage from vessels into perivascular spaces following acute and chronic intraperitoneal treatment with lipopolysaccharide [45]. Sepsis, and the pro-inflammatory cytokines associated with it such as TNF, is known to change BBB integrity and result in vascular leak. Peripheral inflammation increases permeability of cells and molecules, as well as alters tight junctions [46–49]. In a study by Tsao et al., the pro-inflammatory molecule TNF results in BBB dysfunction, and it is well established that TNF is not only higher in TB patients, but correlates to disease severity [49, 50].

Although our previous findings describe the neuropathology associated with peripheral Mtb infection, there is a gap in knowledge on the mechanism of neurotoxicity. We hypothesize that the robust peripheral inflammatory response generated against pulmonary Mtb infection modulates the BBB. This allows for the infiltration of immune cells from the periphery into the brain parenchyma, as well as pro-inflammatory mediators that activate and recruit native glia. Ultimately, this BBB degradation may be a critical initial mechanism that results in the long-term neuropathology associated with the disease. In this study using a relevant guinea pig model of TB, analysis of the major protein constituents of the BBB demonstrated altered expression within vessels. Modulation of BBB proteins resulted in gliosis and the infiltration of immune cells in Mtb-infected animals, localized in the frontal cortex, thalamus, brain stem, and cerebral nuclei. Due to the neuronal loss previously established in this model and its relevancy to neurodegenerative disease, the hippocampus was also evaluated in this study [8]. Through these data, we begin to understand the mechanisms early in the progression of disease which may lead to neurodegeneration and permanent neurological injury, however, this should be investigated further in future studies.

## Results

### Aerosolized Mtb H37Rv and HN878 did not disseminate to the brains of guinea pigs

**Fig 1:**
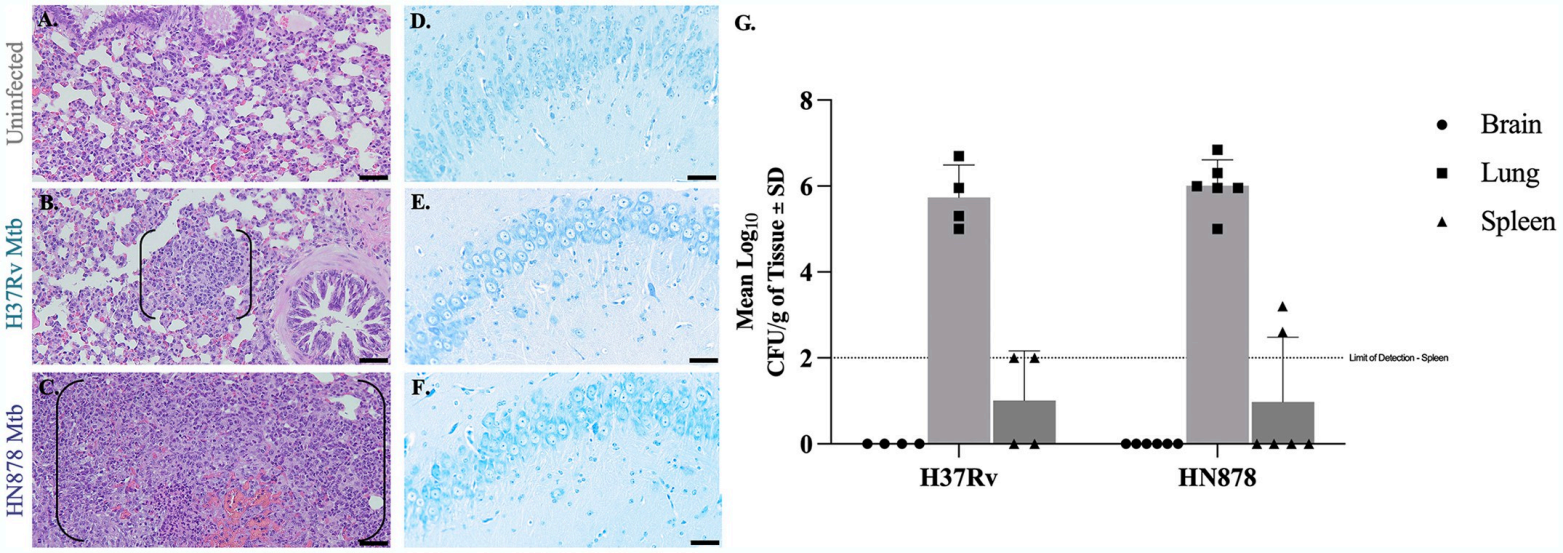
Bacterial dissemination to the brain is not detected despite infection of peripheral tissues. Lesions and bacteria are absent in the brains of guinea pigs infected with Mtb H37Rv and HN878 15 days post-infection. Lung histology shows granulomas (black brackets) in animals infected with Mtb H37Rv (B) and Mtb HN878 (C) but no granuloma formation is seen in uninfected animals (A). Acid-fast staining of brain tissue does not show the presence of bacteria in any brain region; representative images of the hippocampus are given for an uninfected control (D) and an animal infected with Mtb H37Rv (E) and Mtb HN878 (F). Colony-forming unit (CFU) assays indicate bacterial colonies in lung and spleen homogenates, but no bacterial colonies were detected in brain for either Mtb strain. The limit of detection for spleen is 2 log CFU (G). Each bar represents the mean ± SD. (N = 4–6 animals/Mtb infection group). Scale Bar = 100 μm.

Histopathology and organ bacterial burden were used to measure disease severity and bacterial dissemination. Hematoxylin and eosin (H&E) staining of uninfected lung tissue showed normal lung pulmonary structure (Fig 1A). The lungs of animals infected by aerosol with both the laboratory strain Mtb H37Rv (Fig 1B) and clinical strain HN878 (Fig 1C) presented with granulomatous lesions (in brackets). CFU assays confirmed the presence of bacteria in lung homogenate of Mtb H37Rv and HN878 infected animals (Fig 1G). Dissemination of bacteria to the spleen occurred in 50% of animals infected with Mtb H37Rv and in 33% of animals infected with Mtb HN878 (Fig 1G).

In contrast, CFU assays of brain homogenate from infected animals did not contain culturable Mtb bacteria (Fig 1G). Acid-fast staining of whole-brain sagittal tissue sections were negative for the presence of Mtb in infected animals, similar to the uninfected group; representative images of the hippocampus are shown (Fig 1D–1F). From these data, we conclude that infection with aerosolized Mtb H37Rv and HN878 disseminated to the lungs and peripheral organs, but did not reach the brain in this experiment.

### Regionally specific cytosis identified in two animals infected with both a laboratory and clinical Mtb strain

**Fig 2:**
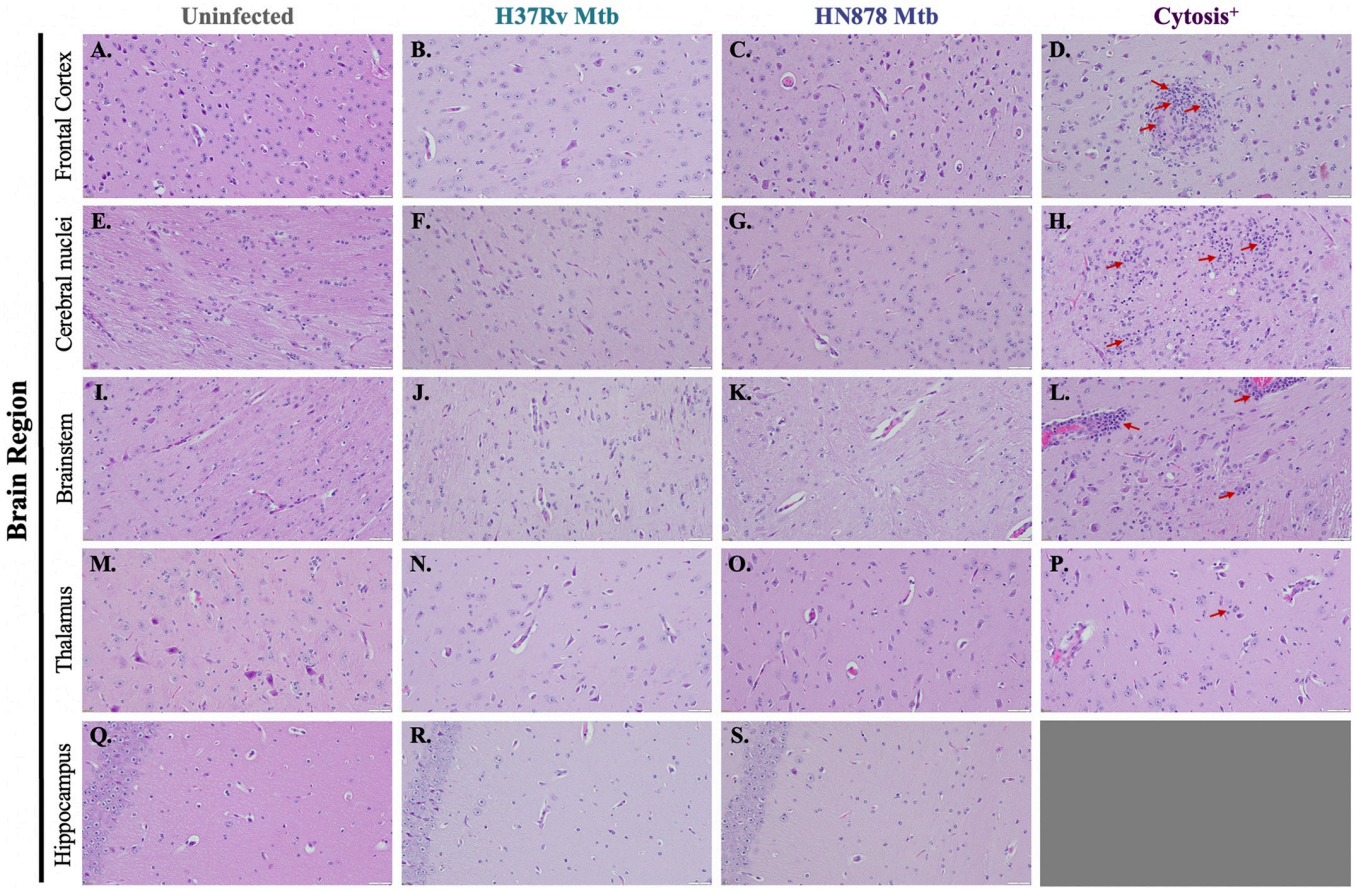
Increased cellular nuclei and changes in tissue morphology in cytosis^+^ animals. Cytosis was identified in the brains of only two Mtb-infected animals by H&E staining. Representative images from the two animals 15 days post-infection in the frontal cortex (A–D), cerebral nuclei (E–H), brain stem (I–L), thalamus (M–P), and hippocampus (Q–S) are shown. No evidence of an abnormal cellular response is seen in any brain region in uninfected controls (A, E, I, M, and Q) nor in the other animals infected with H37Rv (B, F, J, N, and R) and HN878 (C, G, K, O, and S). An intense cellular response, with increased cellular nuclei, is identified in one HN878-infected animal in the frontal cortex (D) and cerebral nuclei (H), and in one Mtb H37Rv-infected animal in the brain stem (L) and thalamus (P). (N = 4–6 animals/Mtb infection group; 2 animals in the cytosis^+^ group). Scale Bar = 50 μm.

Brain tissue from guinea pigs infected with Mtb H37Rv and HN878 was stained with H&E for examination of histopathological changes. Cytosis was identified in one animal infected with Mtb H37Rv, in the brainstem and thalamus, and one animal infected with Mtb HN878, in the frontal cortex and cerebral nuclei (S1 Table). Due to the responses identified in two of the Mtb-infected animals in these regions, and the importance of the hippocampus in neurodegeneration, the frontal cortex, cerebral nuclei, thalamus, brain stem, and hippocampus were selected for further examination. Representative images of the frontal cortex (Fig 2A–2D), cerebral nuclei (Fig 2E–2H), brain stem (Fig 2I–2L), thalamus (Fig 2M–2P) and hippocampus (Fig 2Q–2S) are shown. The cellular response identified in the frontal cortex of the Mtb HN878 infected guinea pig showed a high density of cellular nuclei, indicative of cellular infiltration into that brain region (Fig 2D, red arrows). The response identified in the cerebral nuclei of the Mtb HN878 infected guinea pig had a similarly high density of cellular nuclei (Fig 2H, red arrows). In the brainstem of the Mtb H37Rv infected guinea pig, a high density of cellular nuclei surrounding and within an enlarged blood vessel was identified (Fig 2L, red arrows), similar to the response identified in the thalamus of the Mtb H37Rv infected guinea pig (Fig 2P, red arrows). Within these regions of the neuroparenchyma are multifocal aggregates of moderate numbers of epithelioid macrophages, lymphocytes, and plasma cells was identified in 20% of the Mtb exposed animals. For each of the described brain regions, cytosis to this extent was not seen in the remaining eight Mtb-infected animals tested, nor in the uninfected animals.

### Substantial Iba-1^+^ cellular response in animals infected with both a laboratory and clinical Mtb strain

**Fig 3:**
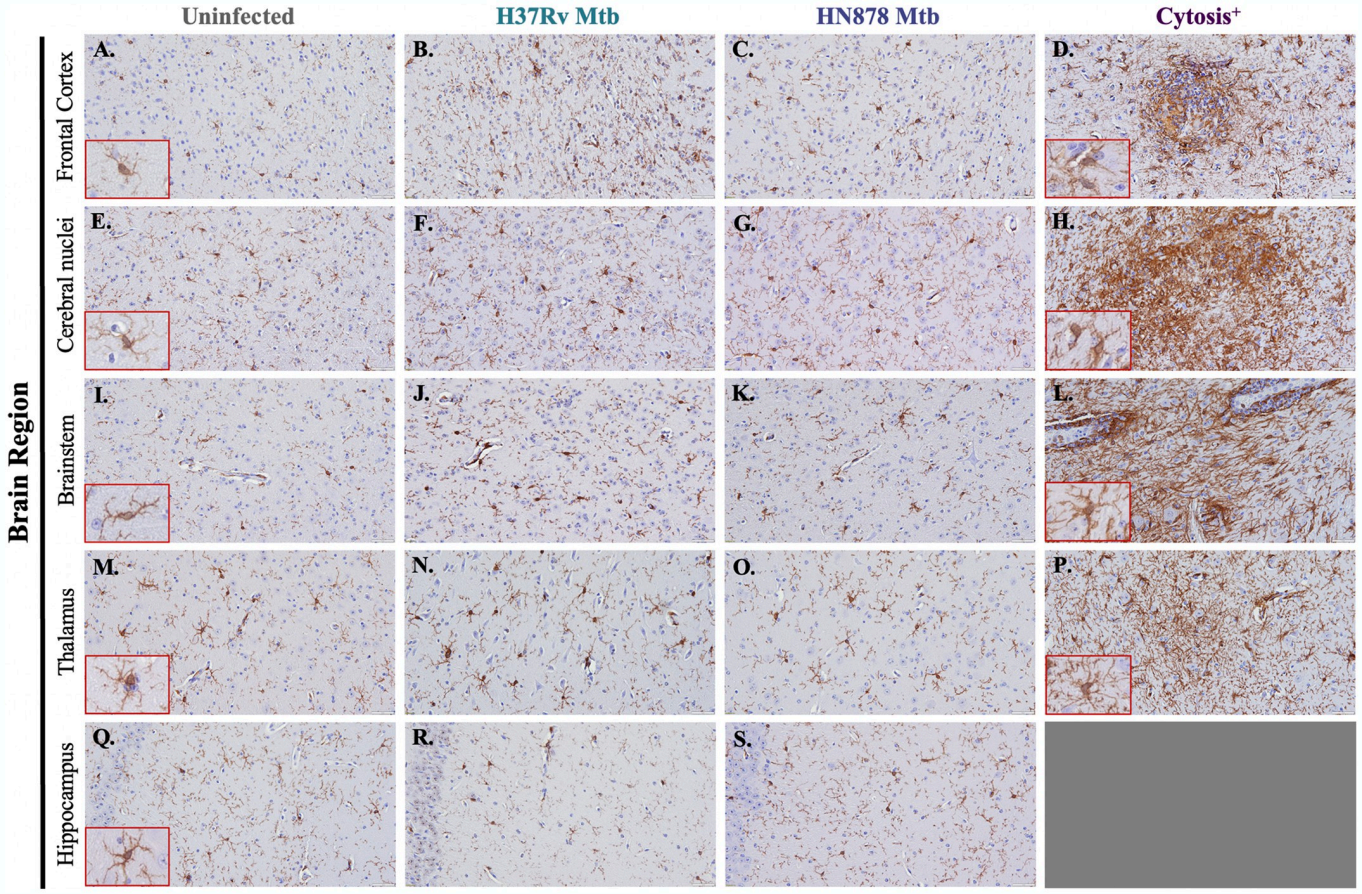
Increased microglial response following Mtb infection that is exacerbated in cytosis^+^ animals. Microgliosis was determined using immunohistochemical staining of guinea pig brain tissue for the microglial cell marker Iba-1. Representative images of Iba-1^+^ microglia 15 days post-infection in the frontal cortex (A–D), cerebral nuclei (E–H), brain stem (I–L), thalamus (M–P), and hippocampus (Q–S) are shown. Inlets were provided to allow for higher magnification to view the glial staining and morphological changes. The number of Iba-1^+^ cells increase in all brain regions in animals infected with Mtb H37Rv (B, F, J, N, and R) and Mtb HN878 (C, G, K, O, and S) compared to uninfected controls (A, E, I, M, and Q). An intense microglial response is identified in one HN878-infected animal in the frontal cortex (D) and cerebral nuclei (H), and in one Mtb H37Rv-infected animal in the brain stem (L) and thalamus (P). (N = 4–6 animals/Mtb infection group; 2 animals in the cytosis^+^ group). Scale Bar = 50 μm.

Glial reactivity in response to pulmonary infection was evaluated using immunohistochemical staining. Representative images of Iba-1^+^ microglial cells in the frontal cortex (Fig 3A–3D), cerebral nuclei (Fig 3E–3H), brain stem (Fig 3I–3L), thalamus (Fig 3M–3P) and hippocampus (Fig 3Q–3S) are shown. Iba-1^+^ microglial cells were seen in the frontal cortex (Fig 3A), cerebral nuclei (Fig 3E), brain stem (Fig 3I), thalamus (Fig 3M), and hippocampus (Fig 3Q) of uninfected animals. Animals infected with both the less virulent H37Rv and hypervirulent HN878 strains of Mtb show increased numbers of Iba-1^+^ cells in those same brain regions (Fig 3B, 3C, 3F, 3G, 3J, 3K, 3N, 3O, 3R and 3S). An exacerbated Iba-1^+^ cellular response, with increased cell number and somal hypertrophy, is seen in the two animals with regional cytosis in the frontal cortex (Fig 3D), cerebral nuclei (Fig 3H), brainstem (Fig 3L), and thalamus (Fig 3P); dense clustering of Iba-1^+^ cells prevented quantitative analysis within these areas. A cellular response to this extent was not identified in the hippocampus, although an increase in Iba-1^+^ cells was still observed following infection. These results are indicative of a progressive Iba-1^+^ cellular response to infection that is intensified in only two of the Mtb-infected animals.

### Reactive astrogliosis in animals infected with both a laboratory and clinical strain of Mtb

**Fig 4:**
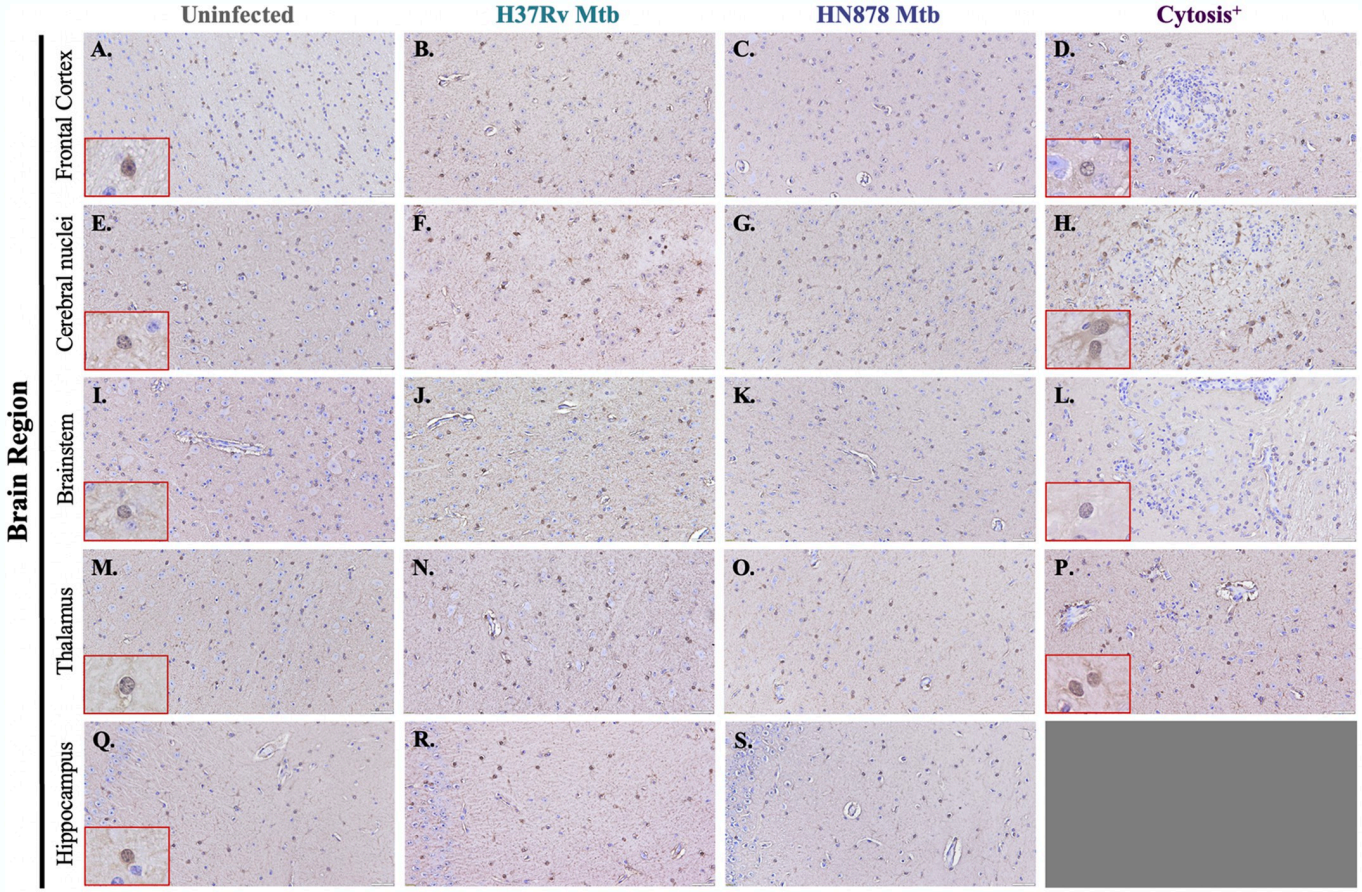
Limited astrocyte proliferation demonstrated following Mtb infection. Immunohistochemical staining of guinea pig brain tissue for the astrocyte marker S100β was performed to determine if proliferation of astrocytes occurred following infection with Mtb. Representative images of S100β^+^ astrocytes 15 days post-infection in the frontal cortex (A–D), cerebral nuclei (E–H), brain stem (I–L), thalamus (M–P), and hippocampus (Q–S) are shown. Inlets were provided to allow for higher magnification to view the glial staining. S100β^+^ cells do not appear to increase in any of the brain regions in animals infected with Mtb H37Rv (B, F, J, N, and R) and Mtb HN878 (C, G, K, O, and S) compared to uninfected controls (A, E, I, M, and Q). Although limited changes in cell number are apparent, cells upregulate S100β in one HN878-infected animal in the frontal cortex (D) and cerebral nuclei (H), and in one Mtb H37Rv-infected animal in the brain stem (L) and thalamus (P). (N = 4–6 animals/Mtb infection group; 2 animals in the cytosis^+^ group). Scale Bar = 50 μm.

In addition to microglia, the astrocytic response was investigated using immunohistochemical and immunofluorescent staining. Representative images of S100β^+^ astrocytes in the frontal cortex (Fig 4A–4D), cerebral nuclei (Fig 4E–4H), brain stem (Fig 4I–4L), thalamus (Fig 4M–4P), and hippocampus (Fig 4Q–4S) are shown. S100β^+^ astrocytes are seen in the frontal cortex (Fig 4A), cerebral nuclei (Fig 4E), brain stem (Fig 4I), thalamus (Fig 4M), and hippocampus (Fig 4Q) of uninfected animals. No change in the number of S100β^+^ cells is seen in animals infected with both the less virulent and hypervirulent strains of Mtb in those same brain regions (Fig 4B, 4C, 4F, 4G, 4J, 4K, 4N, 4O, 4R and 4S). Similarly, animals with regions of cytosis do not show an increase in S100β^+^ cells in the frontal cortex (Fig 4D), cerebral nuclei (Fig 4H), brainstem (Fig 4L), and thalamus (Fig 4P). Animals with cytosis show altered morphology of the S100β^+^ cells, displaying cellular projections with upregulated S100β that are not apparent in uninfected animals (Fig 4D, 4H, 4L and 4P).

**Fig 5:**
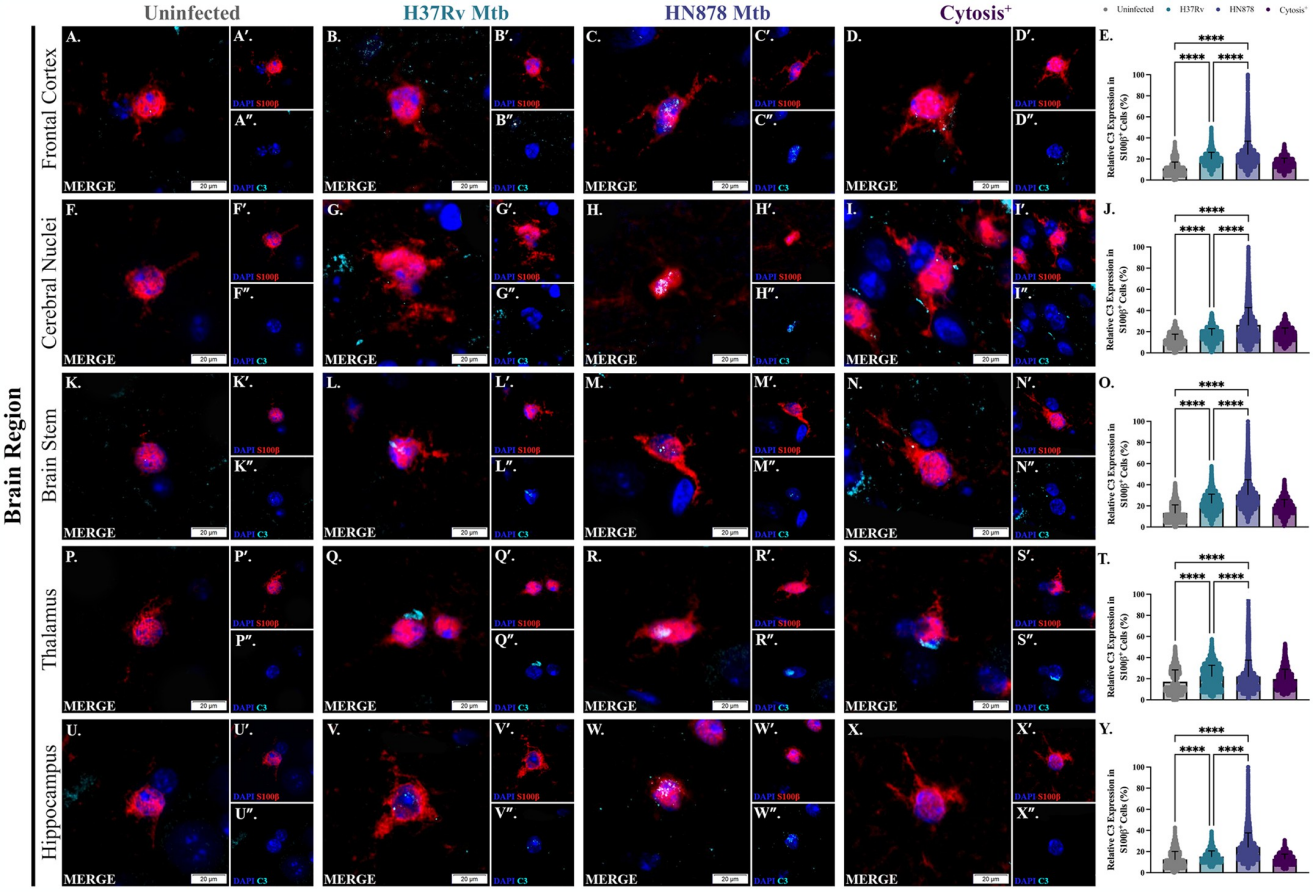
Astrocytes increase production of complement 3 following Mtb-infection. Astrocyte reactivity was analyzed following Mtb exposure for 15 days, as determined by co-localization of DAPI (blue), S100β (red), and C3 (cyan). C3 expression was analyzed in every S100β^+^DAPI^+^ cell present in each brain region for one tissue section per animal. Representative images of the frontal cortex (A–D), cerebral nuclei (F–I), brain stem (K–N), thalamus (P–S), and hippocampus (U–X) 15 days post-infection are shown. Exposure to Mtb H37Rv increased C3 expression, compared to uninfected controls, in the frontal cortex (E), cerebral nuclei (J), brain stem (O), thalamus (T), and hippocampus (Y). Expression is increased in Mtb HN878-infected animals in the frontal cortex (E), cerebral nuclei (J), brain stem (O), thalamus (T), and hippocampus (Y) compared to both H37Rv-infected and uninfected animals. Similarly, C3 is upregulated in astrocytes of cytosis^+^ animals in all brain regions but the hippocampus (E, J, O, T, and Y). Each bar represents the mean ± SD. (N = 4–6 animals/Mtb infection group; 2 animals in the cytosis^+^ group). Nonparametric one-way ANOVA analysis performed; ****p ≤ 0.0001. Scale Bar = 20 μm.

Although Mtb-infected animals do not show substantial proliferation of S100β^+^ cells compared to uninfected animals, the cells present have increased production of C3, which is characterized as an astrocyte with a reactive phenotype. Expression of C3 was quantitatively analyzed within each individual S100β^+^ cell soma. Representative images of immunostaining for C3 in S100β^+^ astrocyte cell bodies in control, Mtb-infected guinea pigs, and the two animals with regional cytosis are depicted in the frontal cortex (Fig 5A–5D), cerebral nuclei (Fig 5F–5I), brain stem (Fig 5K–5N), thalamus (Fig 5P–5S), and hippocampus (Fig 5U–5X). Expression of C3 within each S100β^+^ cell soma was significantly increased in animals infected with Mtb H37Rv in the frontal cortex (p = <0.0001) (Fig 5B and 5E), cerebral nuclei (p = <0.0001) (Fig 5G and 5J), brain stem (p = <0.0001) (Fig 5L and 5O), thalamus (p = <0.0001) (Fig 5Q and 5T), and hippocampus (p = <0.0001) (Fig 5V and 5Y) compared to uninfected controls in those same brain regions (Fig 5A, 5E, 5F, 5J, 5K, 5O, 5P, 5T, 5U and 5Y). Animals infected with Mtb HN878 demonstrate a dramatic increase in C3 expression in the frontal cortex (p = <0.0001) (Fig 5C and 5I), cerebral nuclei (p = <0.0001) (Fig 5H and 5J), brain stem (p = <0.0001) (Fig 5M and 5O), thalamus (p = <0.0001) (Fig 5P and 5T), and hippocampus (p = <0.0001) (Fig 5W and 5Y) compared to uninfected animals. This change in C3 expression in Mtb HN878 infected animals was more significant than in Mtb H37Rv infected animals in the frontal cortex (p = <0.0001) (Fig 5B, 5C and 5E), cerebral nuclei (p = <0.0001) (Fig 5G, 5H and 5J), brainstem (p = <0.0001) (Fig 5L, 5M and 5O), thalamus (p = <0.0001) (Fig 5R and 5T), and hippocampus (p = <0.0001) (Fig 5V, 5W and 5Y). Animals with identified cytosis have increased somal C3 in the frontal cortex (Fig 5D and 5I), cerebral nuclei (Fig 5I and 5J), brain stem (Fig 5N and 5O), and thalamus (Fig 5S and 5T) compared to uninfected controls in those same brain regions (Fig 5A, 5E, 5F, 5J, 5K, 5O, 5P, 5T, 5U and 5Y). Less of a difference is seen between animals with cytosis and those not infected with Mtb in the hippocampus, which is not a region where cytosis was identified (Fig 5U, 5X and 5Y). In all four brain regions, expression was decreased in cytosis^+^ animals (Fig 5D, 5E, 5I, 5J, 5N, 5O, 5S, 5T, 5X and 5Y) compared to those infected with Mtb H37Rv (Fig 5B, 5E, 5G, 5J, 5L, 5O, 5Q, 5T, 5V and 5Y) and Mtb HN878 (Fig 5C, 5E, 5H, 5J, 5M, 5O, 5R, 5T, 5W and 5Y). Overall, these data show the presence of reactive astrocytes, but not cellular proliferation, in Mtb-infected animals with or without cytosis.

### Mtb infection alters aquaporin-4 expression and contact of astrocytic endfeet with vessels

**Fig 6:**
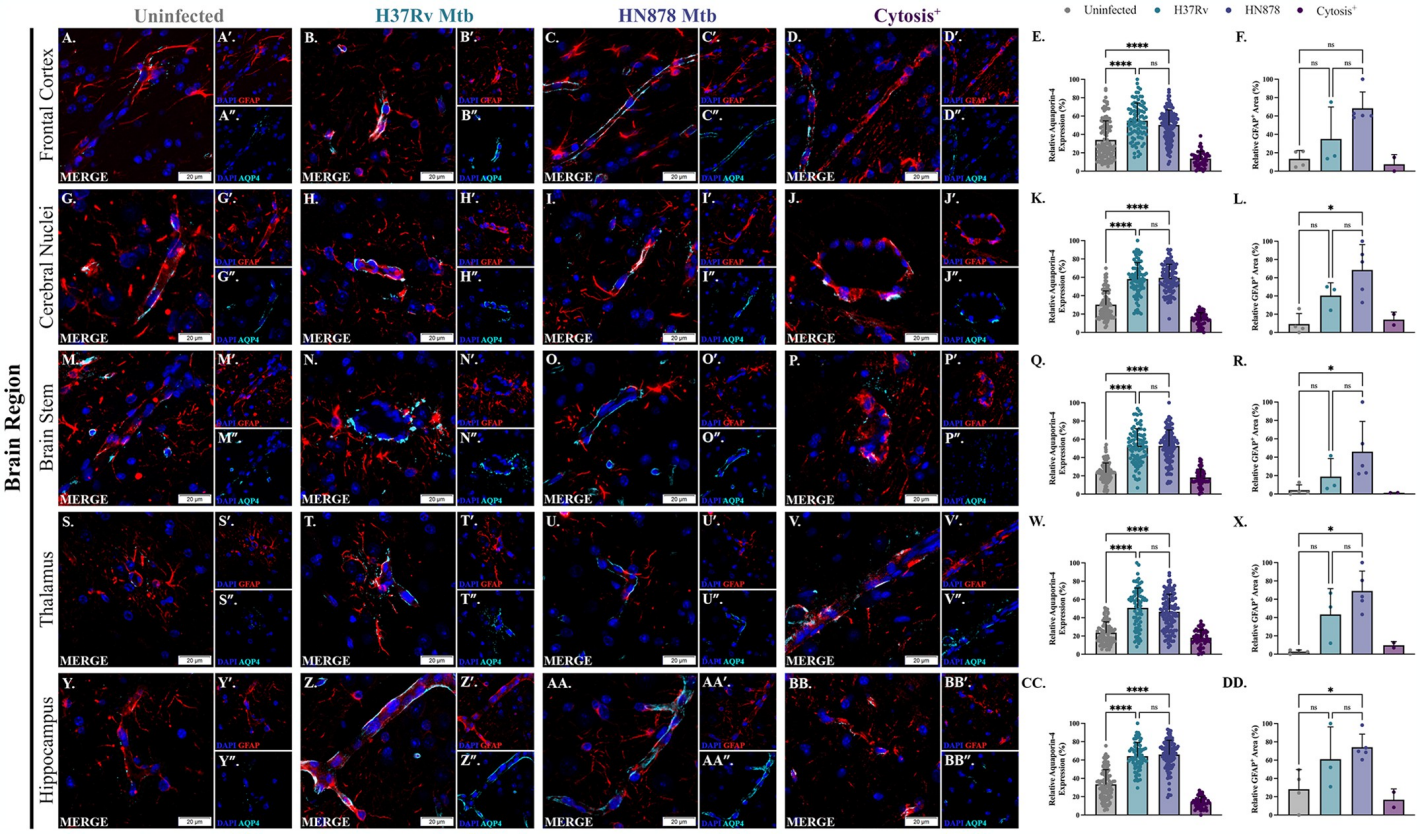
Astrocytes increase contact with vessels and expression of AQP4 following Mtb infection, which is decreased in cytosis^+^ animals. Immunofluorescent staining of guinea pig brain tissue for the astrocytic marker GFAP (red), endfoot protein AQP4 (cyan), and DAPI (blue) in vessels was performed. Representative images of the frontal cortex (A–D), cerebral nuclei (G–J), brain stem (M–P), thalamus (S–V), and hippocampus (Y–BB) 15 days post-infection are shown. Exposure to Mtb H37Rv increased AQP4 expression and endfoot contact, compared to uninfected controls, in the frontal cortex (A, B, and E), cerebral nuclei (G, H, and K), brain stem (M, N, and Q), thalamus (S, T, and W), and hippocampus (Y, Z, and CC). AQP4 expression and contact is increased in Mtb HN878-infected animals in the frontal cortex (A, C, and E), cerebral nuclei (G, I, and K), brain stem (M, O, and Q), thalamus (S, U, and W), and hippocampus (Y, AA, and CC) compared to uninfected animals. Decreased expression of AQP4, and endfoot contact, is found in cytosis^+^ animals in the frontal cortex (D and E), cerebral nuclei (J and K), brain stem (P and Q), thalamus (V and W) and hippocampus (BB and CC). Increased GFAP^+^ area is demonstrated in animals infected with both Mtb H37Rv and HN878, but not cytosis^+^ animals, in the frontal cortex (F), cerebral nuclei (L), brain stem (R), thalamus (X), and hippocampus (DD). Each bar represents the mean ± SD. (N = 4–6 animals/Mtb infection group; 2 animals in the cytosis^+^ group). Nonparametric one-way ANOVA analysis performed; * = p ≤ 0.05, **** = p ≤ 0.0001. Scale Bar = 20 μm.

Astrocytes, identified as GFAP^+^ cells, and the water channel protein AQP4, which is found on astrocytic endfeet, were identified using immunofluorescence microscopy; protein expression of AQP4 in association with vessels was quantitatively analyzed. Representative images of vessels with AQP4 and GFAP^+^ astrocyte processes in uninfected controls, Mtb-infected guinea pigs, and the two animals with regional cytosis are depicted in the frontal cortex (Fig 6A–6D), cerebral nuclei (Fig 6G–6J), brain stem (Fig 6M–6R), thalamus (Fig 6S–6V) and hippocampus (Fig 6Y–6BB). Expression of AQP4 within vessels was significantly increased in animals infected with Mtb H37Rv compared to controls in the frontal cortex (p = <0.0001) (Fig 6A, 6B and 6E), cerebral nuclei (p = <0.0001) (Fig 6G, 6H and 6K), brainstem (p = <0.0001) (Fig 6M, 6N and 6Q), thalamus (p = <0.0001) (Fig 6S, 6T and 6W), and hippocampus (p = <0.0001) (Fig 6Y, 6Z and 6CC). Similarly, animals infected with Mtb HN878 demonstrated significantly increased AQP4 expression compared to uninfected animals in the frontal cortex (p = <0.0001) (Fig 6A, 6C and 6E), cerebral nuclei (p = <0.0001) (Fig 6G, 6I and 6K), brainstem (p = <0.0001) (Fig 6M, 6O and 6Q), thalamus (p = <0.0001) (Fig 6S, 6U and 6W), and hippocampus (p = <0.0001) (Fig 6Y, 6AA and 6CC). There was no strain-dependent difference in expression in all regions, including the frontal cortex (p = 0.4937) (Fig 6B, 6C and 6E), cerebral nuclei (p = >0.9999) (Fig 6H, 6I and 6K), brainstem (p = >0.9999) (Fig 6N, 6O and 6Q), thalamus (p = >0.9137) (Fig 6T, 6U and 6W), and hippocampus (p = >0.9999) (Fig 6Z, 6AA and 6CC). Expression of AQP4 in cytosis^+^ animals decreased compared to not only Mtb H37Rv-infected and Mtb HN878-infected animals, but also uninfected controls in the frontal cortex (Fig 6A–6E), cerebral nuclei (Fig 6G–6K), and hippocampus (Fig 6Y–6CC). A similar trend is seen between cytosis^+^ animals and animals infected with both strains in the brain stem (Fig 6N–6Q) and thalamus (Fig 6T–6W), but no change was seen when compared to uninfected controls in these two brain regions.

Concurrently, GFAP^+^ astrocyte processes appear to increase contact in animals infected with Mtb H37Rv (Fig 6B, 6H, 6N, 6T and 6Z) and HN878 (Fig 6C, 6I, 6O, 6U and 6AA) compared to uninfected controls (Fig 6A, 6G, 6M, 6S and 6Y). Retraction of processes from vessels is observed in cytosis^+^ animals (Fig 6D, 6J, 6P, 6V and 6BB). These changes in contact of the astrocytic endfeet with vessels is supported by greater GFAP^+^ area in the brain regions of Mtb-infected animals, which is indicative of cellular hypertrophy and increased process volume. An insignificant, but trending, increase in GFAP^+^ area is found in animals infected with Mtb H37Rv compared to uninfected controls in the frontal cortex (p = >0.9999) (Fig 6F), cerebral nuclei (p = 0.4648) (Fig 6L), brain stem (p = 0.6783) (Fig 6R), thalamus (p = 0.2377) (Fig 6X), and hippocampus (p = 0.4390) (Fig 6DD). Animals infected with Mtb HN878 show a significant increase in GFAP^+^ area compared to uninfected animals in the cerebral nuclei (p = 0.0179) (Fig 6L), brain stem (p = 0.0244) (Fig 6R), thalamus (p = 0.0168) (Fig 6X), and hippocampus (0.0393) (Fig 6DD), although no significant difference occurs in the frontal cortex (p = 0.0553) (Fig 6F). Cytosis^+^ animals have a GFAP^+^ area similar to controls, as no difference is observed in the frontal cortex (Fig 6F), cerebral nuclei (Fig 6L), brain stem (Fig 6R), thalamus (Fig 6X), and hippocampus (Fig 6DD). Overall, Mtb infection increases GFAP^+^ area, astrocyte process contact with vessels, and AQP4 expression compared to controls, but this response appears to be decreased in cytosis^+^ animals.

### Modulation of the blood-brain barrier following infection with Mtb

**Fig 7:**
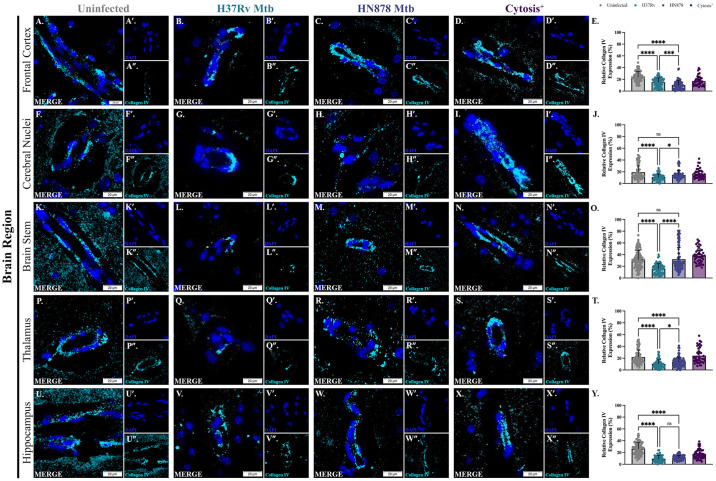
Collagen IV is modulated following infection with Mtb. Immunofluorescent staining of guinea pig brain tissue for DAPI (blue) and collagen IV (cyan) in vessels was performed. Representative images of the frontal cortex (A–D), cerebral nuclei (F–I), brain stem (K–N), thalamus (P–S), and hippocampus (U–X) 15 days post-infection are shown. Exposure to Mtb H37Rv and HN878 decreased collagen IV expression compared to uninfected controls in the frontal cortex (E), cerebral nuclei (J), brain stem (O), thalamus (T), and hippocampus (Y). Decreased expression of collagen IV in cytosis^+^ animals is found in the frontal cortex (E) and hippocampus (Y), but not the cerebral nuclei (J), brain stem (O), and thalamus (T). Each bar represents the mean ± SD. (N = 4–6 animals/Mtb infection group; 2 animals in the cytosis^+^ group). Nonparametric one-way ANOVA analysis performed; *p ≤ 0.05, ***p ≤ 0.001, and ****p ≤ 0.0001. Scale Bar = 20 μm.

Immunofluorescence microscopy was utilized to evaluate the expression of BBB-associated proteins within blood vessels; this included matrix collagen IV and the tight junction protein claudin V. Representative images of immunostaining for collagen IV in control, Mtb-infected guinea pigs, and the two animals with regional cytosis are depicted for the frontal cortex (Fig 7A–7D), cerebral nuclei (Fig 7F–7I), brain stem (Fig 7K–7N), thalamus (Fig 7P–7S), and hippocampus (Fig 7U–7X). In the frontal cortex, expression of collagen IV within blood vessels was significantly decreased in animals infected with Mtb H37Rv compared to controls (p = <0.0001) (Fig 7A, 7B and 7E), and in animals infected with Mtb HN878 (p = <0.0001) (Fig 7A, 7C and 7E). Expression was further decreased in Mtb HN878 infected animals compared to H37Rv (p = 0.0010). Animals with identified cytosis show decreased expression of collagen IV compared to uninfected animals in this brain region (Fig 7A, 7D and 7E). Vessel collagen IV expression in the cerebral nuclei was significantly decreased in Mtb H37Rv-infected animals compared to uninfected controls (p = <0.0001) but not HN878-infected (p = 0.3031) (Fig 7F–7H and 7J); H37Rv-infected animals had significantly lower expression than HN878 (p = 0.0186). No change was identified between animals with cytosis and those uninfected (Fig 7F, 7I and 7J). In the brain stem, a decrease in expression of collagen IV occurred in animals infected with Mtb H37Rv compared to uninfected animals (p = <0.0001) (Fig 7K, 7L and 7O), although no significant difference was detected in animals infected with Mtb HN878 compared to controls (p = 0.2927) (Fig 7K, 7M and 7O). No change was detected in animals with regional cytosis compared to uninfected controls (Fig 7K, 7N and 7O). Similar to the frontal cortex, the thalamus showed decreased collagen IV in infected animals of both strains compared to uninfected controls (p = <0.0001 for both strains) (Fig 7P–7R and 7T); animals exposed to Mtb H37Rv had statistically lower expression than Mtb HN878 (p = 0.0357). No change occurred between animals with cytosis and uninfected animals (Fig 7P, 7S and 7T). This trend in expression was seen in the hippocampus as well, although animals with cytosis had a decreased expression of collagen IV compared to uninfected animals (Fig 7U, 7X and 7Y), but not as low as the other animals infected with Mtb H37Rv (p = <0.0001) (Fig 7V, 7X and 7Y) nor HN878 (p = <0.0001) (Fig 7W–7Y).

**Fig 8:**
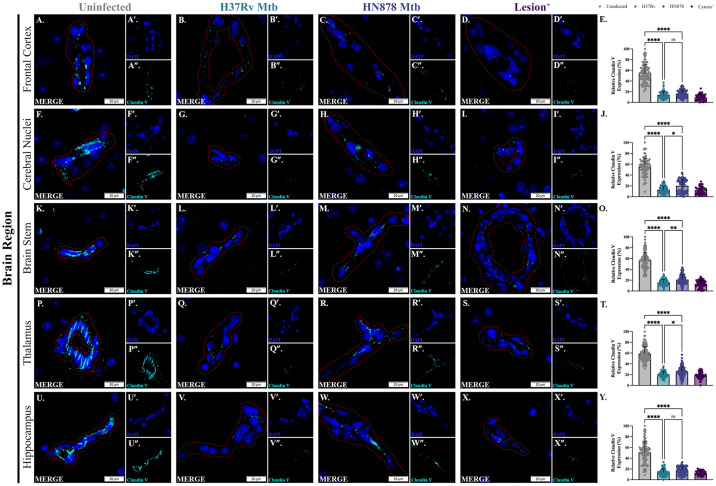
Claudin V is decreased following infection with Mtb. Immunofluorescent staining of guinea pig brain tissue for DAPI (blue) and claudin V (cyan) in vessels was performed. Representative images of the frontal cortex (A–D), cerebral nuclei (F–I), brain stem (K–N), thalamus (P–S), and hippocampus (U–X) 15 days post-infection are shown. Exposure to Mtb H37Rv and HN878 decreased claudin V expression compared to uninfected controls in the frontal cortex (E), cerebral nuclei (J), brain stem (O), thalamus (T), and hippocampus (Y). Decreased expression of claudin V in cytosis^+^ animals is found in the frontal cortex (E), cerebral nuclei (J), brain stem (O), thalamus (T), and hippocampus (Y) as well. Each bar represents the mean ± SD. (N = 4–6 animals/ Mtb infection group; 2 animals in the cytosis^+^ group). Nonparametric one-way ANOVA analysis performed; *p ≤ 0.05, **p ≤ 0.01, and ****p ≤ 0.0001. Scale Bar = 20 μm.

Expression of claudin V, a component of tight junctions, within vessels was also evaluated. Representative images of immunostaining for claudin V in control, Mtb-infected guinea pigs, and the two animals with regional cytosis are depicted for the frontal cortex (Fig 8A–8D), cerebral nuclei (Fig 8F–8I), brain stem (Fig 8K–8N), thalamus (Fig 8P–8S), and hippocampus (Fig 8U–8X). In the frontal cortex, animals infected with Mtb H37Rv (Fig 8B and 8E) and Mtb HN878 (Fig 8C and 8E) showed decreased expression of claudin V compared to uninfected controls (p = <0.0001 for both) (Fig 8A and 8E). No strain difference was observed (p = 0.2688). Decreased expression in Mtb-infected animals compared to uninfected animals was also seen in the cerebral nuclei (p = <0.0001 for both), although expression in animals infected with Mtb HN878 was not as low as H37Rv and a strain-specific difference is observed (p = 0.0264) (Fig 8F–8H and 8J). The same trend in expression was seen in the brain stem (Fig 8K–8O) and thalamus (Fig 8P–8T). In these two regions, and in the hippocampus, Mtb-infected animals had significantly lower expression than uninfected controls (p = <0.0001 for all). Strain-specific differences were observed in the brain stem (p = 0.0047) (Fig 8O) and thalamus (p = 0.0266) (Fig 8T). In the hippocampus, claudin V expression was not significantly different between strains (Fig 8U–8Y) (p = 0.1361). In all five brain regions, a decrease in claudin V expression is observed in the cytosis^+^ animals compared to uninfected controls (Fig 8E, 8J, 8O, 8T and 8Y). Analysis of these proteins demonstrate that Mtb infection modulates collagen IV expression in a strain-dependent manner. The observed expression of claudin V was decreased, regardless of strain, in Mtb-infected and cytosis^+^ animals in this experiment.

### Infiltration of peripheral immune cells into the brain of two Mtb-infected animals

**Fig 9:**
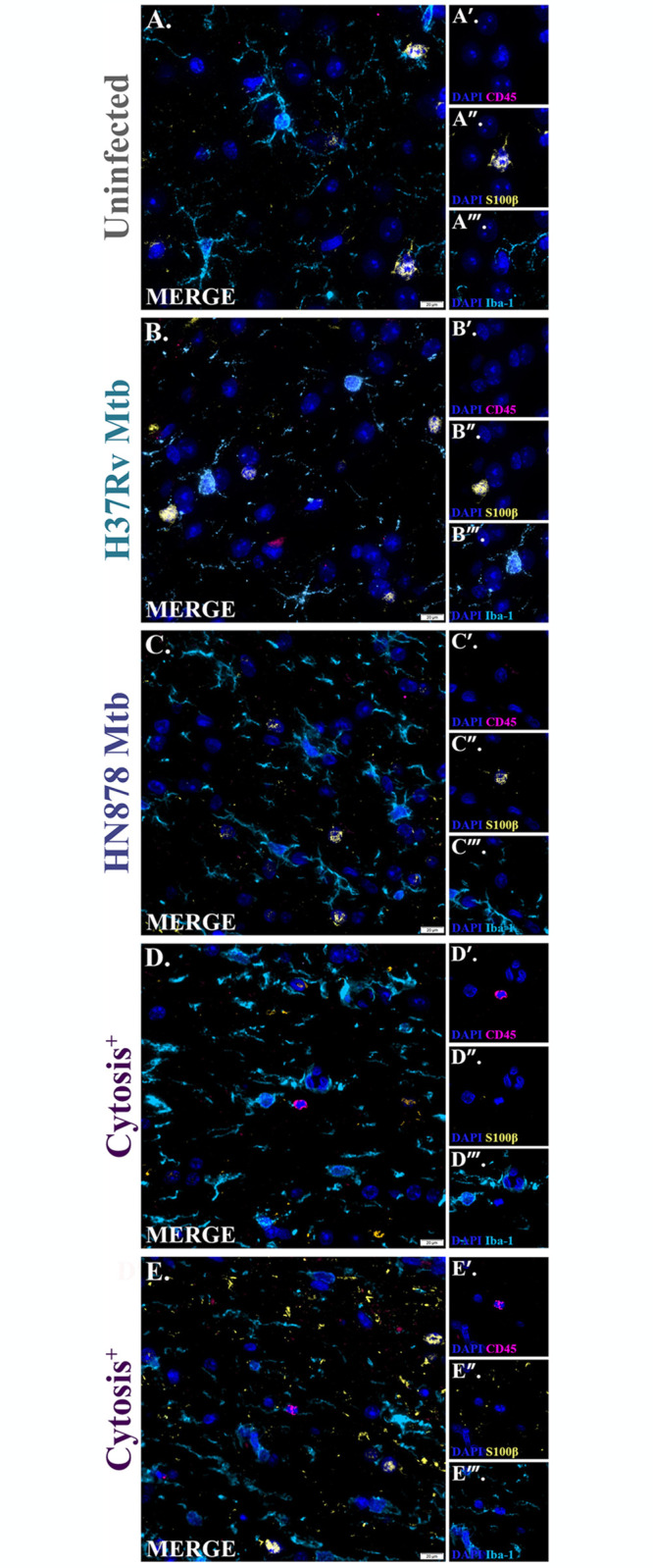
Peripheral immune cells identified in cytosis^+^ animals. Immunofluorescent staining of guinea pig brain tissue for CD45 (pink), S100β (yellow), Iba-1 (cyan), and DAPI (blue) for identification of peripheral immune cells. Representative images 15 days post-infection are shown. Exposure to Mtb H37Rv (B) and HN878 (C) did not show an abnormal population of cells, similar to uninfected animals (A). Alternatively, CD45^high^S100β^low^Iba-1^low^ peripheral immune cells are identified in cytosis^+^ animals (D and E). (N = 4–6 animals/Mtb infection group; 2 animals in the cytosis^+^ group). Scale Bar = 20 μm.

Immunofluorescence microscopy was used to phenotypically profile cells within each brain region to determine if immune cells from the periphery were infiltrating into the brain. Although glia can express lymphocyte common antigen cluster of differentiation 45 (CD45), a protein tyrosine phosphatase that is expressed on all nucleated hematopoietic cells, it is expressed at low to intermediate levels. Therefore, peripheral immune cells were identified in this study as CD45 high expressing, Iba-1 low expressing, and S100β low expressing cells (CD45^hi^Iba-1^low^S100β^low^). An increase of phenotypically CD45^hi^Iba-1^low^S100β^low^ cells are detected in the brains of animals with cytosis (Fig 9D and 9E) but not in the other animals infected with Mtb (Fig 9B and 9C) nor in uninfected controls (Fig 9A).

## Discussion

The neurological effects of TB have only recently been described, clinically and in laboratory models. Our current knowledge includes the progressive activation of glia that culminates in behavior changes, aggregated misfolded proteins, and hippocampal neuronal loss in response to peripheral Mtb infection in a guinea pig model [8]. Although the neuropathologies that may contribute to TB-associated cognitive dysfunction have been described, the mechanism of neurotoxicity has not yet been elucidated. Our previous findings demonstrate that neuroinflammation and pathological changes occur without detectible bacteria in the brains of Mtb-infected animals, highlighting mechanisms of cellular stress in response to chronic systemic inflammation during Mtb infection [8]. Meningococcal diseases and bacterial infections, including those caused by Mtb, demonstrate that bacterial and host-mediated BBB permeability occurs [51]. Previous work from Brilha et al. has shown that Mtb decreases expression of BBB proteins *in vitro* [41]. Other studies have also revealed that systemic inflammation results in BBB dysfunction which exacerbates neuroinflammation. We, therefore, hypothesized that pulmonary Mtb infection without dissemination to the brain modulates the BBB, allowing for the infiltration of peripheral immune cells and, potentially, neurotoxic molecules into the brain that initiate neuroinflammatory responses. In our current study, a guinea pig model with the intention of reducing bacterial dissemination to the brain was used. A short study duration of 15 days post-infection was chosen in order to evaluate the neurological changes that occur early in the progression of disease, prior to the glial and neuronal changes identified in our previously published findings [8]. We demonstrate in a small cohort of animals for the first time, to our knowledge, that peripheral infection with both a laboratory and a clinical strain of Mtb, without evidence of CNS infection, alters the expression of proteins critical to the integrity and function of the BBB *in vivo* in combination with cellular reactivity.

Bacterial diversity contributes to the variable outcomes of clinical TB cases. Although host factors play a role in the progression of pulmonary infection into clinical disease, it has become increasingly apparent that the bacteria is genetically diverse [52]. Even bacterial stocks of the same strain between laboratories have genetic variability [53]. Studies utilizing Mtb sometimes disregard strain differences, often due to experimental constraints, which limits the translational capacity of the findings. For this reason, our relevant guinea pig model of TB disease consisted of aerosolized infection with approximately 80 CFU of two different bacterial strains, Mtb H37Rv and HN878. As the first Mtb isolate to be genomically characterized, Mtb H37Rv is one of the most widely used strains in laboratory research [54, 55]. Experimentation using another strain, the virulent clinical isolate W-Beijing HN878, is becoming more common. Although HN878 has a similar doubling time as H37Rv during the first 14 days of infection, it is considered a hypervirulent bacterial strain due to decreased animal survival compared to other laboratory strains, likely because of reduced T-helper 1 cell responses [56]. While strains like H37Rv do not cause granulomatous lesions in inherently Mtb-resistant murine models, Mtb HN878 induces granulomas in the lung with a pathology found in humans, including a core of macrophages surrounded by lymphocytes, as well as B-cell lymphoid follicles and germinal centers [57]. Some clinical strains have also proven to disseminate more readily to the CNS than laboratory strains like Mtb H37Rv, making it imperative that additional strains be utilized when studying the BBB during infection [58]. Examining the neurological effects of infection with two strains, both a laboratory and a clinical strain, helps account for the innate variability between strains in our study.

This study consisted of three-month-old, outbred Dunkin Hartley guinea pigs. Our use of a guinea pig *in vivo* model, whose pathology and cellular response to Mtb closely mimics that of humans, is the best current laboratory model of neuropathology caused by peripheral Mtb infection and enhances the translational potential of our findings [59–61]. Young animals were chosen for this study, as it is well-established that natural aging results in BBB dysfunction, neuroinflammation, and the accumulation of neurotoxic proteins [39, 40, 62]. The Dunkin Hartley guinea pig is known to exhibit aged-related pathology as early as five-months-old, in both the brain and systemic tissues [63–65]. By using young animals, we eliminate possible age-related impact, although our findings of neuropathology caused by pulmonary Mtb infection may exacerbate underlying neurological changes in aged models.

In our model, granulomatous lesions were identified in the lungs of animals 15 days post-infection (dpi) (Fig 1), without signs of morbidity or mortality, or significant weight loss, irrespective of infectious strain (data not shown). Although cytosis was identified in only two of these Mtb-infected animals, no granulomatous lesions were found in the brain as commonly seen with TBM. The gold standard bacterial colony forming unit (CFU) assay did not detect bacteria in the brain tissue of any animals despite CFUs present in lung and bacterial dissemination to the spleen occurred in half of the animals infected with Mtb H37Rv and one third of the animals infected with Mtb HN878. This data was supported by acid-fast staining of brain tissue, which did not identify any bacilli in Mtb-infected animals (Fig 1). The combination of these three findings, determined utilizing well-established experimental methods, indicate that neurological changes are likely not induced by live bacteria infiltrating the CNS, despite evidence that bacterial dissemination occurred to extrapulmonary organs. Although we provide evidence that detectable bacterial dissemination to the brain did not occur through multiple methods, we acknowledge the limitations of these experiments. It is understood within the field that these methods lack sensitivity and may not detect small quantities of disseminated bacteria. While whole bacteria, both culturable and non-culturable, are not identified in the brain, our methods also do not account for bacterial components that maintain a high degree of immunogenicity. Additionally, a larger sample size is necessary to determine with certainty if this model of TB reduces bacterial dissemination to the brain.

Despite the lack of granulomatous lesions in the brain, one animal from each experimental group, infected with Mtb H37Rv or HN878, showed areas with an abnormal cellular response, or cytosis. Brain regions included the frontal cortex, thalamus, brain stem, and cerebral nuclei (S1 Table). Histopathological staining identified the presence of increased numbers of immune cells, including lymphocytes (Fig 2). Identification of these areas of cytosis determined the brain regions studied for the remainder of the manuscript. The hippocampus was also selected, as it is an area susceptible to neurodegenerative effects and previously implicated in TB neuropathology [8].

Crosstalk between microglia and astrocytes is a known modulator of neuroinflammation. Typically considered the first cell to respond in the brain, microglia readily migrate and activate in response to stress, like trauma or contact with pathogens, sometimes as quickly as a couple of hours [66]. Under pathological conditions, microglial activation has been implicated in BBB dysfunction [67, 68]. Because of these roles, microgliosis was assessed by immunohistochemistry for the detection of Iba-1. Increased Iba-1^+^ cells are seen in guinea pigs infected with both Mtb H37Rv and HN878, and a substantial microglial response is demonstrated in the two cytosis^+^ animals (Fig 3). The visual appearance of the microglia in Mtb-infected animals suggests an activated phenotype, with shorter processes, cell hypertrophy, and an ameboid-like morphology; microglia with longer processes are identified in control animals [69–71]. These observations suggest that pulmonary Mtb infection promotes the polarization of microglia into a reactive morphology in the animals that were undergoing brain cytosis. Notably, activated microglia are known to secrete Il-1α, TNF, and C1q, which are sufficient in inducing a reactive astrocytic phenotype [10]. Microglia also upregulate the production of reactive oxygen species (ROS) and pro-inflammatory molecules like IL-1β when activated [72]. Unfortunately, Iba-1 is not solely a microglial marker but is also expressed at low levels on peripheral macrophages [73]. Activated microglia and peripheral macrophages also share an ameboid-like morphology, making it increasingly challenging to differentiate between these cell types. It must be considered that the cellular response, especially in cytosis^+^ animals, may also consist of peripheral macrophages.

Despite a substantial microglial response, proliferation or the recruitment of S100β^+^ astrocytes in any brain region of animals infected with Mtb, with or without identified cytosis, was lacking (Fig 4); however, this does not consider the phenotype of the cells present. The exact function of astrocytic C3 is context-dependent, but it has been shown that reactive astrocytes upregulate C3 in neuroinflammatory and neurodegenerative diseases, demonstrating C3 as a marker of astrogliosis [10, 74–76]. Upon further investigation, C3 expression within S100β^+^ cell soma was increased in all brain regions of Mtb-infected animals compared to uninfected controls, and this response was amplified in Mtb HN878-infected guinea pigs (Fig 5). Such data indicates that the astrocytes in these regions display a more reactive phenotype. This change in reactivity, but not cell number, makes sense in the context of cellular activation versus proliferation. While astrocytes respond to stimuli, such as mediators produced by nearby microglia, within hours, it can take days for these cells to expand their populations properly [77]. Temporal limitations in the study and no evidence of culturable bacteria in the brain may not have allowed sufficient time for astrocyte proliferation.

Limited changes in C3 expression were seen in the hippocampus of cytosis^+^ animals compared to controls, although it should be noted that cytosis was not identified in any animals within this region. Alternatively, increased C3 was seen in cytosis^+^ animals compared to uninfected controls in the other four brain regions (Fig 5), suggesting that the extent of astrogliosis may play a role in BBB integrity and the recruitment of immune cells; an increased sample size is necessary for certainty. Polarized microglia, as seen in Mtb-infected animals, can secrete IL-1β which stimulates the release of the pro-inflammatory molecules C-C motif chemokine ligand 2 (CCL2), C-C motif chemokine ligand 20 (CCL20), and C-X-C motif chemokine ligand 2 (CXCL2) by activated astrocytes. These three mediators are known to encourage the migration of immune cells into the brain [78]. Additionally, microglial IL-1β represses the production of sonic hedgehog (SHH) by astrocytes, which results in reduced BBB integrity and decreased tight junction proteins [78]. The identification of reactive astrocytes that upregulate C3 in Mtb-infected animals and in animals with cytosis unveils a potential mechanism for the modulation of the BBB seen in these animals that should be investigated further.

Interestingly, although no change in astrocyte cell number is observed qualitatively, more GFAP^+^ processes are present in Mtb-infected animals, along with an increase in total GFAP area (Fig 6). In cases of neuroinflammation, reactive astrocytes, characterized as having longer, branched processes, increase contact with the vasculature, which results in increases in total cell area [79]. The C3-secreting, phenotypically reactive astrocytes identified in Mtb-infected animals are increasing contact with the vasculature, which is supported by our data showing more GFAP^+^ processes in association with blood vessels (Fig 6). This further establishes the formation of a reactive population of astrocytes in response to pulmonary Mtb infection.

Increased expression of AQP4 was found in animals infected with Mtb H37Rv and HN878 (Fig 6). Expression of AQP4 was located within the endfeet of astrocytes with a reactive phenotype, as is common in neuroinflammatory states [80]. The exact function of AQP4 is disputed. Some studies exert that AQP4 expression leads to BBB changes, and is itself pro-inflammatory [81]. In a study by Zhou et al., AQP4 knockout mice opened tight junctions, downregulated GFAP expression, and increased vascular permeability [82]. In contradiction, others state that while AQP4 is involved in brain maintenance, alterations in this protein alone do not result in BBB leakage, immunoreactivity, or modified structure of microvessels [83]. Thus, we must consider the impact of altered AQP4 expression within the context of the complex BBB. A decrease in AQP4 expression is found in cytosis^+^ animals compared to controls (Fig 6). This is likely due to the retraction of astrocytic endfeet, as glia can withdraw their endfeet during neuroinflammatory states [84]. Retracted endfeet may play a role in reducing barrier tightness and allowing for immune cell infiltration in areas with cytosis, as *in vivo* and *in vitro* studies show that astrocytes can reduce leukocyte traffic through the formation of TJs by upregulating claudins and junctional adhesion molecules [34]. It is possible that process retraction reduced stimulation of TJs by astrocytes in these two cytosis^+^ brain regions.

Glia are linked to the BBB in other ways, and that is through enzymatic regulation of proteins. Collagen levels are controlled by MMPs, which are endopeptidases that degrade the extracellular matrix [85]. Collagen IV, in particular, is regulated by matrix metalloproteinase-2 (MMP-2) and matrix metalloproteinase-9 (MMP-9) in the brain. These enzymes are tightly controlled, at both the level of transcription and post-translation, to prevent unnecessary damage. MMPs are often upregulated with age or in diseases, like AD, and it is proposed that these changes are due to a pro-inflammatory brain phenotype. MMPs are also highly implicated in BBB dysfunction [85]. Reactive astrocytes and microglia upregulate transcription of MMP-2 and MMP-9, and the pro-inflammatory mediators they secrete, like TNF, can enhance MMP transcription [86–89]. Activated glia also produce ROS, resulting in proteolytic cleavage or disruption of thiol interactions that activate MMPs [89, 90]. By morphology and secretory phenotype, it has been established that reactive glia are present in Mtb-infected animals; these cells are likely upregulating MMPs and secreting mediators that indirectly modulate enzymatic activity. Together, MMPs can degrade collagen IV in the vasculature, which may result in decreased expression, as seen in our study (Fig 7).

Similarly, MMPs are involved in the loss of TJ proteins, like the primary constituent claudin V, which significantly decreases following Mtb exposure (Fig 8). Like collagen IV, claudin V is also degraded by MMP-9. Studies show that activation of MMPs during pathological events degrades tight junction proteins like claudin V, resulting in increased BBB permeability [91, 92]. This is demonstrated in other infection models; the bacteria involved in numerous meningococcal diseases, such as *Neisseria meningitidis*, can induce BBB changes through MMP-mediated proteolytic cleavage of the tight junction proteins [51, 93]. MMP-9 has also been directly implicated in peripheral immune cell infiltration into the brain [88]. Glial activation may be leading to increased activity of MMPs that are degrading matrix and TJ proteins. This mechanism seems likely given that *in vitro* studies have shown that Mtb modulates MMP activity [41].

The brain is considered an immunologically privileged organ. Peripheral immune cells, like T cells and macrophages, are found near the healthy brain in meningeal and perivascular spaces, but their existence in the parenchyma is limited [94]. Evidence shows that peripheral immune cells are more common in the resting brain than previously described, especially dendritic and memory T cells. However, the presence of infiltrating immune cells is still considered an event of the compromised brain [94–96]. Differential expression of surface proteins is a common method to differentiate immune cell populations. While glia express cluster of differentiation 45 (CD45), it is in low to intermediate levels compared to peripheral immune cells. This makes CD45 expression, in combination with other markers, a common technique used to identify neurological versus peripheral immune cells [97–99]. This study identified peripheral immune cells as S100β^low^Iba-1^low^CD45^hi^; S100β^hi^CD45^low^ astrocytes and Iba-1^hi^CD45^low^ microglia were excluded. Based on these parameters, we detected the presence of a peripheral immune cell population that is not evident in uninfected animals nor those infected with Mtb without cytosis (Fig 9). This is indicative of immune cell infiltration from the peripheral circulation.

Increased BBB permeability has been correlated with the invasion of immune cells into the brain. It is partially why this event is documented in the aged brain and cases of neurodegenerative disease, including AD and PD. The function of peripherally invading immune cells has not been completely defined, and some evidence of neuroprotective roles exists, but they are primarily described as having neurotoxic functions. Invading cells activate glia and sustain neuroinflammation, leading to the degeneration of neurons and decline of cognitive function [94, 100, 101]. Although we distinguish the presence of peripheral immune cells based on surface expression, phenotyping centered on cell surface markers is incredibly complex. Cells demonstrate standard expression profiles but can upregulate or downregulate surface proteins, especially during inflammation and disease; even CD45 is modulated in inflammatory environments [102, 103]. Surface expression and morphology indicate that the cells we have identified are of peripheral origin, but we also acknowledge that an Iba-1^+^CD45^low^ microglial population has been recently described. Cells of this phenotype exist in small populations, and are, to our current knowledge, associated with the diseased brain. This suggests that if such a microglial population exists in our TB model, they may still contribute to the pathogenesis attributed to pulmonary infection and should be further evaluated [104]. Glial markers in combination with CD45 expression alone do not allow us to properly categorize what types of cells we identify in the brains of cytosis^+^ animals. While we detect the infiltration of immune cells into the brain, further experimentation is necessary to classify these cells properly.

Despite demonstrating that pulmonary Mtb infection modulates BBB proteins, and we expect this will lead to vascular leak, experimentation is needed to establish the extent of barrier permeability fully. Future studies with a larger sample size that employ optimized permeability assays are necessary to definitively determine BBB modulation, if vascular leakage into the brain is occurring and, if so, what size biomolecules can penetrate. That being said, even established protocols utilizing BBB tracers as a measure of permeability cannot account for small molecules and ions [105]. This is pertinent given the small size of neurotoxic microbial components, like cell wall proteins and bacterial DNA, that may be penetrating the brain from the peripheral circulation. Permeability assays are, therefore, only sometimes a reliable way of examining barrier integrity, although they may be beneficial for future studies [105].

These results describe changes to major BBB constituents that we correlate to gliosis and peripheral immune cell infiltration without evidence of bacterial dissemination to the brain. In response to Mtb exposure, microglia and astrocyte reactivity is demonstrated in multiple brain regions. We correlate these phenotypic changes to increased AQP4 expression and astrocyte process contact with the vasculature. Additionally, we identify decreased expression of TJ proteins and altered collagen levels that can be attributed to Mtb infection. Ultimately, infiltration of peripheral immune cells is identified in animals with progressive neuropathology. Due to the sample size, the main limitation of this study, additional experimentation is necessary to fully characterize the pathological changes occurring in the Mtb-infected animals that demonstrate cellular infiltration. Our data identifies modifications to the BBB shown, by others in other pathological states, to contribute to barrier dysfunction, providing valuable information on the mechanism of TB-associated neurotoxicity. These findings, which have never been identified in the context of peripheral TB disease, unveil potential mechanisms of cellular stress that justify further comprehensive investigation of the phenomena described.

## Materials and methods

### Animals and sample collection

Three-month-old, female, outbred Dunkin–Hartley guinea pigs (Elm Hill, USA) were used in this study. They were housed in a biosafety level 3 laboratory at the Colorado State University Laboratory Animal Resources facility accredited by the American Association for Accreditation of Laboratory Animal Care (AAALAC). Animals were pair housed under constant temperature and humidity conditions (21° ± 2 °C temperature and 30 ± 5% humidity). A 12-hour light/12-hour dark cycle was used, and animals had *ad libitum* access to standard pelleted food and water. Animals were monitored using a clinical scoring system for signs of morbidity and weighed weekly by laboratory staff for the entirety of the experiment. Experiments were performed in accordance with the National Research Council’s Guide for the Care and Use of Laboratory Animals and were approved by the Institutional Animal Care and Usage Committee (IACUC) at Colorado State University. At the time of euthanasia, guinea pigs were administered 50 mg/kg of ketamine and 5 mg/kg of xylazine via intramuscular injection for anesthetic induction. Under terminal anesthesia, guinea pigs were euthanized by intraperitoneal overdose of pentobarbital. Tissues were collected for histopathology by fixing in 10% buffered formalin or stored at -80°C for subsequent homogenization and quantification of Mtb colony forming units.

### Mtb aerosol exposure

This study utilized two strains of Mtb: the laboratory strain Mtb H37Rv, which is considered less virulent, and the clinical strain Mtb HN878, a hypervirulent strain. Culture stocks of *Mycobacterium tuberculosis* (Mtb) strain H37Rv (TMC #102, Trudeau Institute) and HN878 (Clinical Isolate W210, CSU, Fort Collins, CO) were collected at an OD600 nm between 0.8 and 1.0 and frozen at -80°C in Proskauer-Beck liquid medium containing 0.05% Tween-80. Titer was determined and bacteria were diluted in sterile water to 2 × 10^6^ colony forming units (CFU)/mL. Animals were exposed to an aerosolized dose of Mtb for twelve minutes, calibrated to deliver 50–100 bacilli per animal. The Glas-Col Airborne whole-body exposure apparatus was used to infect the animals in one run per strain, and approximately 80 CFU of Mtb were delivered by aerosol to each animal. Four animals were infected with Mtb H37Rv, and six animals were infected with HN878. Each run contained a single guinea pig for euthanasia and necropsy 24 hours after exposure to confirm Mtb delivery to the lungs. Animals were exposed to Mtb for a total of 15 days post-infection (dpi). Four uninfected animals were exposed to sterile water using the same procedure in the Glas-Col device.

### Bacterial burden/CFU counts

For confirmation of bacterial enumeration at 24 hours, the lung was homogenized in 30 mL of PBS and plated on 150 x 50mm petri dishes containing 7H11 with 10% OADC, 10 μg/mL cycloheximide, and 50 μg/mL of carbenicillin. For quantification of bacterial dissemination at the end of the study period, lung, spleen, and brain were collected, weighed, and homogenized in PBS. The entirety of the brain homogenate for each animal was diluted 1:10 followed by serial dilutions of tissue homogenate in PBS. Dilutions were plated as stated above and all agar plates were incubated at 37 °C + 5% CO_2_. Following 6–8 weeks of incubation, CFU’s were counted, and CFU’s per gram of tissue were calculated.

### Tissue processing, embedding, and histological staining

Brains and visceral organs were fixed in 10% neutral buffered formalin at room temperature for at least 48 hours. Tissues were processed using a Leica TP1020 Automatic Benchtop Tissue Processor and embedded in paraffin wax (Cancer Diagnostics, Cat #: EEPAR56). Tissues were sectioned on a Thermo Scientific HM 325–2 Manual Microtome at 5μm thickness and mounted on positively charged glass slides (Superfrost Plus, Cancer 232 Diagnostics, Cat #: 4951) for staining and analysis. One section per animal was deparaffinized and stained with hematoxylin (Cancer Diagnostics, Cat#: #HTV-4) and eosin (Cancer Diagnostics, Cat#: #ETV) (H&E) for determination of histopathological changes. Brain sections were sent to the Colorado State University’s Veterinary Diagnostic Laboratory for acid-fast staining to determine if bacterial dissemination to the brain occurred. An Mtb-positive lung tissue slide was also acid-fast stained to confirm the validity of the staining procedure.

### Immunohistochemical staining

Immunohistochemical stains were performed on whole brain sagittal sections. To deparaffinize the tissue sections, slides were heated for 20 minutes at 60°C followed by a series of incubations in xylene and graded ethanol (xylene, 1 part xylene to 1 part 100% EtOH, 100% EtOH, 95% EtOH, 70% EtOH, 1.0 M TBS) for 5 minutes each. Heat- and chemical-induced antigen retrieval was performed on the tissue by incubating in 0.01 M sodium citrate (pH = 6.0) for 20 minutes at 95°C. Removal of endoperoxides was performed through incubation in 0.3% hydrogen peroxide for 30 minutes at room temperature. Wash steps and tissue permeabilization was performed using 2% bovine serum albumin (BSA) and 2% Triton-X in 1.0 M TBS. Tissue was blocked in 10% goat or donkey serum diluted in 1.0 M TBS for 1 hour at room temperature. After being diluted to their optimized concentrations, primary antibodies were incubated on the tissue at 4°C overnight.

Astrocytes were identified using a rabbit anti-S100 calcium-binding protein β (S100β) antibody at a 1:750 concentration (Abcam, Cat #: ab41548). Microglia were identified using a goat anti-ionized calcium binding adaptor molecule 1 (Iba-1) antibody at a 1:400 concentration (Abcam, Cat #: ab5076). Washing was performed using 2% BSA in 1.0 M TBS before incubation with the secondary antibody at a 1:250 concentration for 1 hour at room temperature. The following secondary antibodies were used for the astrocyte and microglial stains, respectively: goat anti-rabbit secondary antibody (Vector Labs, Cat #: BA-1000) and donkey anti-goat secondary antibody (Jackson ImmunoResearch, Cat #: 705-065-147).

Following secondary incubation, tissue was incubated an additional hour in ABC mix from an ABC HRP peroxidase detection kit (Vector Laboratories, Cat #: PK-4000). An ImmPACT DAB Substrate, Peroxidase (HRP) Kit (Vector Laboratories, Cat #: sk-4105) was used as chromogen, with a fixed chemical reaction period for each antigen of interest. Slides were counterstained with hematoxylin (Thermo Fisher Scientific, Cat #: 7231) and bluing reagent (Cancer Diagnostics, Cat #: FX2107). Stained tissue was preserved under a glass coverslip with mounting medium (Richard-Allen Scientific, Cat #: 4112) and stored at room temperature. Whole tissue images were taken for analysis using an Olympus BX53 microscope with an Olympus DP70 camera using an Olympus UPlanSApo 20x objective (N.A. = 0.75). Representative images were taken using an Olympus BX53 microscope with an Olympus DP70 camera using an Olympus UPlanSApo 20x objective (N.A. = 0.75) and Olympus UPlanFL N 40x objective (N.A. = 0.75). See Table 1.

**Table 1:** Immunohistochemistry antibodies.

Primary Antibody and Concentration Used	Primary Antibody Catalog Number	Secondary Antibody and Concentration Used	Secondary Antibody Catalog Number
Rabbit anti-S100β 1:750	Abcam Cat #: ab41548	Goat anti-rabbit IgG (H+L) 1:250	Vector Labs Cat #: BA-1000
Goat anti-Iba-1 1:400	Abcam Cat #: ab5076	Donkey anti-goat IgG (H+L) 1:250	Jackson ImmunoResearch Cat #: 705-065-147

### Immunofluorescent staining

Whole brain sagittal sections were deparaffinized by heating slides for 20 minutes at 60°C followed by incubation in xylenes and graded ethanol (xylene, 1 part xylene to 1 part 100% EtOH, 100% EtOH, 95% EtOH, 70% EtOH, 1.0 M TBS) for 5 minutes each. Heat- and chemical-induced antigen retrieval was performed by incubating tissue in 1X EDTA buffer (1mM EDTA disodium salt dihydrate, 0.05% Tween; pH 8.0) for 20 minutes at 95°C. Tissue was washed with 0.05 M TBS and blocked using 2% donkey and/or goat serum in TrisA (0.2% Triton-X in 1.0 M TBS) for 1 hour at room temperature. After being diluted to their optimized concentrations, primary antibodies were incubated on the tissue at 4°C overnight.

Blood-brain barrier integrity was analyzed by detecting collagen IV, claudin V, and aquaporin-4 (AQP4). Collagen IV was identified using an anti-Collagen IV antibody at a 1:100 concentration (NOVUS, Cat #: NB120-6586SS) and a donkey anti-rabbit Alexa Fluor 647 secondary antibody (Invitrogen, Cat #: A31573). Claudin V was identified using an anti-Claudin 5 polyclonal antibody at a 1:500 concentration (Invitrogen, Cat #: 34–1600) and a donkey anti-rabbit Alexa Fluor 647 secondary antibody (see above). AQP4 was identified using an anti-Aquaporin-4 monoclonal antibody at a 1:100 concentration (ABclonal, Cat #: A11210) and a donkey anti-rabbit Alexa Fluor 647 secondary antibody (see above). For visualizing astrocytes, three antibodies were used: a chicken anti-Glial fibrillary acidic protein (GFAP) antibody at a 1:200 concentration (Aves Labs, Cat #: GFAP), a rabbit anti-Complement 3 (C3) antibody at a 1:100 concentration (Abcam, Cat #: ab181147), and a mouse anti-S100 calcium-binding protein β (S100β) antibody at a 1:500 concentration (Abcam, Cat #: ab212816). The astrocyte stain used the following secondary antibodies: goat anti-chicken Alexa Fluor 488 (Invitrogen, Cat #: A11039), donkey anti-rabbit Alexa Fluor 647 (Invitrogen, Cat #: A31573), and donkey anti-mouse Alexa Fluor 555 (Invitrogen, Cat #: A31570). Infiltrating immune cells were identified by co-staining for CD45, Iba-1, and S100β. To do this, CD45 was identified using an anti-CD45 (IH-1) monoclonal antibody at a 1:100 concentration (NOVUS Biologicals, Cat #: NB100-65362) and donkey anti-mouse Alexa Fluor 555 secondary antibody at a 1:500 concentration (Invitrogen, Cat #: A31570). Microglia were identified using a goat anti-ionized calcium binding adaptor molecule 1 (Iba-1) antibody at a 1:50 concentration (Abcam, Cat #: ab5076) and Donkey anti-goat Alexa Fluor 647 secondary antibody at a 1:500 concentration (Invitrogen, Cat #: A21447). S100β was identified using an anti-S100β antibody at a 1:500 concentration (Abcam, Cat #: ab212816) and Donkey anti-rabbit Alexa Fluor 488 secondary antibody at a 1:500 concentration (Southern Biotech, Cat #: 6441–30). A detailed table outlining the antibodies and concentrations used is included below.

Four 10-minute wash steps (1.0 M TBS) were followed by incubation with the secondary antibodies at 1:500 concentrations for at least 1 hour at room temperature in the dark. Tissue was washed three times for five minutes each (1.0 M TBS), and stained with Hoechst (Thermo Scientific, Cat #: 62249) diluted 1:2000 in PBS for three minutes followed by three additional washes (1.0 M TBS). Slides were coverslipped with Prolong Gold Anti-fade mounting medium (Cell Signaling Technology, Cat #: 9071), allowed to harden for 24 to 48 hours at room temperature, and then stored at 4°C in the dark. Whole-slide images were acquired using an Olympus BX63 fluorescence microscope equipped with a motorized stage and Hamamatsu ORCA-flash 4.0 LT CCD camera using a 20x Olympus X Apochromat air objective air objective (N.A. = 0.80). All slides, irrespective of experimental group, were imaged on the same day with the same exposure time per channel. Representative images were captured using an Olympus BX63 fluorescence microscope equipped with a motorized stage and Hamamatsu ORCA-flash 4.0 LT CCD camera using a 40x Olympus X278 Apochromat air objective air objective (N.A. = 0.80). Red blood cells were removed from representative images. See Table 2.

**Table 2:** Immunofluorescent antibodies.

Protein of Interest:	Primary Antibody and Concentration Used	Primary Antibody Catalog Number	Secondary Antibody and Concentration Used	Secondary Antibody Catalog Number
Collagen IV	Rabbit anti-Collagen IV 1:100	NOVUS Cat #: NB120-6586SS	Donkey anti-rabbit Alexa Fluor 647 1:500	Invitrogen Cat #: A31573
Claudin V	Rabbit anti-Claudin V 1:500	Invitrogen Cat #: 34–1600	Donkey anti-rabbit Alexa Fluor 647 1:500	Invitrogen Cat #: A31573
AQP4	Rabbit anti-Aquaporin-4 1:100	ABclonal Cat #: A11210	Donkey anti-rabbit Alexa Fluor 647 1:500	Invitrogen Cat #: A31573
GFAP	Chicken anti-Glial fibrillary acidic protein (GFAP) 1:200	Aves Labs Cat #: GFAP	Goat anti-chicken Alexa Fluor 488 1:500	Invitrogen Cat #: A11039
C3	Rabbit anti-Complement 3 antibody 1:100	Abcam Cat #: ab181147	Donkey anti-rabbit Alexa Fluor 647 1:500	Invitrogen Cat #: A31573
S100β	Mouse anti-S100 calcium-binding protein β (S100β) 1:500	Abcam Cat #: ab212816	Donkey anti-mouse Alexa Fluor 555 1:500	Invitrogen Cat #: A31570
S100β	Rabbit anti-S100 calcium-binding protein β (S100β) 1:500	Abcam Cat #: ab41548	Donkey anti-rabbit Alexa Fluor 488 1:500	Southern Biotech Cat #: 6441–30
CD45	Mouse anti-CD45 (IH-1) antibody 1:100	NOVUS Biologicals Cat #: NB100-65362	Donkey anti-mouse Alexa Fluor 555 1:500	Invitrogen Cat #: A31570
Iba-1	Goat anti-Iba-1 1:50	Abcam Cat #: ab5076	Donkey anti-goat Alexa Fluor 647 1:500	Invitrogen Cat #: A21447

### Immunofluorescent analysis

Whole slide images of Collagen IV, Claudin V, and AQP4 stained by immunofluorescence were analyzed. For each slide, regions of interest (ROIs) were drawn around individual blood vessels of various sizes and orientations within the tissue. ROIs were evenly distributed across the following five brain regions: frontal cortex, hippocampus, thalamus, cerebral nuclei, and brainstem. At least twenty vessels per brain region were analyzed, and care was taken to exclude red blood cells and other abnormalities or artifacts from the analysis. Mean gray intensity of the proteins within each vessel were quantified using manual thresholding on the Count and Measure function of Olympus CellSens software (v1.18). Reactive astrocytes were quantified by analyzing complement 3 expression within S100β^+^ astrocytes. This was performed by using manual thresholding on the Count and Measure function of Olympus CellSens software (v1.18) to identify S100β^+^DAPI^+^ cells, which were then converted into individual ROIs. Manual thresholding was then used to quantify the fluorescence intensity of C3 within each S100β^+^ cell ROI. Adaptive thresholding on the Count and Measure function of CellSens was used to select for GFAP^+^ area for each brain region. Percent relative expression was calculated for each protein of interest by determining the minimum (min) and maximum (max) quantifications for the data set. Each raw quantification [106] for that brain region received the following calculation: [(raw–min)/(max–min)*100].

### Statistical analysis

All data is presented as mean +/− SD unless otherwise specified. A ROUT (Q = 1%) outlier test was performed on all data to identify potential outliers, which were removed from the data set. Differences between experimental groups were analyzed using a nonparametric one-way ANOVA with Dunn’s multiple comparisons test. Statistical analysis was completed using Graphpad Prism. Significance is denoted throughout the manuscript as * = p ≤ .05, ** = p ≤ 0.01, *** = p ≤ 0.001, and **** = p ≤ 0.0001.

## Supporting information

S1 Table Degree of cytosis in various brain regions for each guinea pig infected with Mtb H37Rv and Mtb HN878. (PDF)
