# Supplementary material for: Immune cell infiltration and modulation of the blood-brain barrier in a guinea pig model of tuberculosis: Observations without evidence of bacterial dissemination to the brain
Source: PLoS One. 2024 Dec 31;19(12):e0307577. doi: 10.1371/journal.pone.0307577 (PMC11687776; doi:10.1371/journal.pone.0307577)
Supplement: S1 Table — (PDF) [file pone.0307577.s001.pdf]

Supplementary Table 1: Cytosis identified in two Mtb-infected animals

Animal:	Mtb Strain:	Frontal Cortex	Cerebral Nuclei	Brainstem	Thalamus	Hippocampus
Guinea Pig #1	H37Rv	-	-	+	+	-
Guinea Pig #2	H37Rv	-	-	-	-	-
Guinea Pig #3	H37Rv	-	-	-	-	-
Guinea Pig #4	H37Rv	-	-	-	-	-
Guinea Pig #5	HN878	-	-	-	-	-
Guinea Pig #6	HN878	+	+	-	-	-
Guinea Pig #7	HN878	-	-	-	-	-
Guinea Pig #8	HN878	-	-	-	-	-
Guinea Pig #9	HN878	-	-	-	-	-
Guinea Pig #10	HN878	-	-	-	-	-

One guinea pig infected with Mtb H37Rv (N = 4) showed cytosis in the brain stem and the thalamus. One guinea pig infected with Mtb HN878 (N = 6) showed cytosis in the frontal cortex and cerebral nuclei. No animals presented with cytosis in the hippocampus, which was evaluated further due to the role this brain region plays in neurodegenerative disease.
